# Treatment of HFpEF beyond the SGLT2-Is: Does the Addition of GLP-1 RA Improve Cardiometabolic Risk and Outcomes in Diabetic Patients?

**DOI:** 10.3390/ijms232314598

**Published:** 2022-11-23

**Authors:** Martina Belli, Lucy Barone, Alfonso Bellia, Domenico Sergi, Dalgisio Lecis, Francesca Romana Prandi, Marialucia Milite, Chiara Galluccio, Saverio Muscoli, Francesco Romeo, Francesco Barillà

**Affiliations:** 1 Division of Cardiology, Department of Systems Medicine, Tor Vergata University, 00133 Rome, Italy; 2 Cardiovascular Imaging Unit, San Raffaele Scientific Institute, 20132 Milan, Italy; 3 Department of Systems Medicine, Tor Vergata University, 00133 Rome, Italy; 4 Department of Cardiology, Mount Sinai Hospital, Icahn School of Medicine at Mount Sinai, New York, NY 10029, USA; 5 Department of Departmental Faculty of Medicine, UniCamillus-Saint Camillus International University of Health and Medical Sciences, 00131 Rome, Italy

**Keywords:** heart failure with preserved ejection fraction, phenotypes, SGLT2 inhibitors, GLP1 receptor agonists

## Abstract

Heart failure with preserved ejection fraction (HFpEF) is a common clinical syndrome frequently seen in elderly patients, the incidence of which is steadily increasing due to an ageing population and the increasing incidence of diseases, such as diabetes, hypertension, obesity, chronic renal failure, and so on. It is a multifactorial disease with different phenotypic aspects that share left ventricular diastolic dysfunction, and is the cause of about 50% of hospitalizations for heart failure in the Western world. Due to the complexity of the disease, no specific therapies have been identified for a long time. Sodium-Glucose Co-Transporter 2 Inhibitors (SGLT2-Is) and Glucagon-Like Peptide Receptor Agonists (GLP-1 RAs) are antidiabetic drugs that have been shown to positively affect heart and kidney diseases. For SGLT2-Is, there are precise data on their potential benefits in heart failure with reduced ejection fraction (HFrEF) as well as in HFpEF; however, insufficient evidence is available for GLP-1 RAs. This review addresses the current knowledge on the cardiac effects and potential benefits of combined therapy with SGLT2-Is and GLP-1RAs in patients with HFpEF.

## 1. Introduction 

HFpEF is not a well-defined clinical entity, but a multifactorial disease. It is an amalgam of cardiovascular (CV), metabolic, renal, and geriatric conditions [1,2], with different phenotypic aspects that have joint left ventricular diastolic dysfunction (LVDD). The impairment of left ventricular (LV) compliance leads to an increase in cardiac chamber stiffness and elevated filling pressures, which are responsible for the onset and worsening of subjective and objective clinical signs of HF. HFpEF is the cause of about 50% of hospital admissions for heart failure in the Western world [3]. The diagnosis of HFpEF is based on the presence of signs and symptoms of HF with normal LV ejection fraction (EF). 

Type 2 diabetes mellitus (T2DM) and heart failure (HF) frequently occur together [4,5]. The prevalence of T2DM in patients with HF ranges from 40% to 50%. The prevalence of HF in patients with T2DM is estimated to be ≈20% [6,7,8,9]. In patients with HF, mortality is higher in patients with T2DM than in patients without concomitant T2DM [10,11,12].

The pathophysiology of HF in diabetic patients is complex. “Diabetic cardiomyopathy”, defined as ventricular systolic or diastolic dysfunction in the absence of other causes (for example, coronary artery disease), is an increasingly recognized entity [13]. Several potential mechanisms contributing to the development of HF in diabetes include activation of the renin-angiotensin-aldosterone system (RAAS); oxidative stress; inflammation; intracellular calcium homeostasis alterations; increased formation of advanced glycation end products; and myocardial energy substrate mismatch, including increased utilization of free fatty acids, decreased glucose utilization, and increased oxygen consumption, leading to energy deficiency [14,15,16,17,18].

Especially in view of the emerging cardiometabolic phenotypes (obesity, type 2 diabetes mellitus, and hypertension), GLP-1RA and SGLT2-Is are receiving increasing attention as potential therapeutic strategies in HFpEF because of their glucose-lowering, anti-inflammatory, and anti-remodeling effects. Indeed, GLP-1RAs and SGLT2-Is have been shown to reduce the risk of CV events in T2DM patients because of several proposed protective mechanisms [19]. Therefore, the combination of these drugs may further improve CV and renal outcomes, especially in HFpEF patients with high or very high cardio-metabolic risk.

In this review, we summarise the current evidence on the potential benefit of GLP-1 RAs and SGLT2-Is in patients with HFpEF, especially in those with high cardio-nephro-metabolic risk.

## 2. Phenotyping Aspects and the Burden of Cardio-Nephro-Metabolic Risk in HFpEF

Multiple mechanisms lead to HFpEF, including dysfunction in atrial and ventricular active relaxation, increased passive stiffness, and reduction in arterial compliance. Moreover, the presence of endothelial oxidative stress is associated with a decreased nitric oxide (NO) bioavailability, which causes vasoconstriction and enhances a pro-inflammatory and pro-thrombotic state. The cardiac remodeling is affected by changes in the composition and structure of the extracellular matrix with fibrillar collagen deposition. In such a scenario, the LV diastolic dysfunction is the final disease expression of heterogeneous phenotypes and clinical presentations [20].

**Figure 1:**
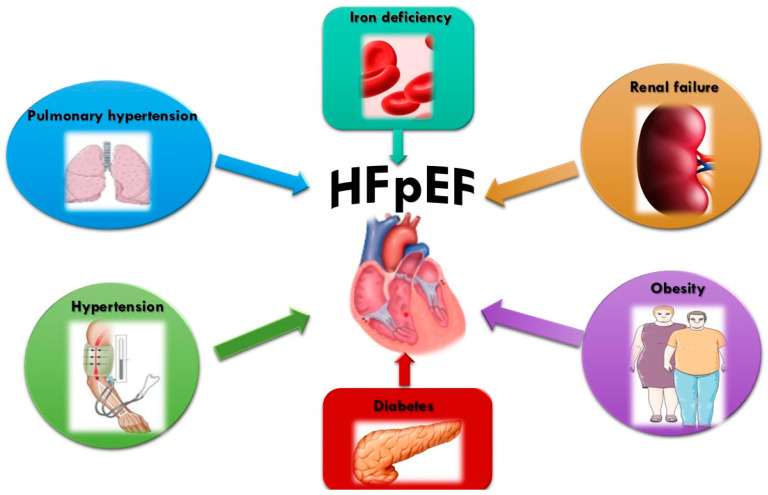
The influence of comorbidities on the development of HFpEF. Iron deficiency, diabetes mellitus, obesity, renal failure, hypertension, and pulmonary hypertension increase the risk of HFpEF through structural and functional cardiac change, and systemic inflammation.

Hypertension is the steadfast underpinning of HFpEF. Ageing, obesity, pulmonary hypertension (PH), and coronary artery disease affect HFpEF presentation and progression [21,22]. Their presence defines the different phenotypes seen in patients with HFpEF. These phenotypes share some comorbid conditions, such as iron deficiency, chronic obstructive pulmonary disease (COPD), diabetes, and chronic kidney disease (CKD) [23] (Figure 1).

**Ageing Phenotype:** Age is a major risk factor for HFpEF [24,25]. Many studies have shown that the incidence of HFpEF is very high in elderly and very elderly subjects (>80 years of age) [26,27]. Ageing is associated with many changes, most importantly neurohormonal dysregulation and proinflammatory state, which promote increased arterial stiffness and hypertension. By increasing systolic arterial pressure, arterial stiffening imposes an excessive load on the heart, leading to LVDD [28,29]. Clinical events are more frequent when HFpEF is associated with severely reduced LV compliance, but, in this stage, it may longer be susceptible to therapy. The lack of therapeutic success of a lot of drugs in patients with HFpEF is because the time of treatment may be earlier at the stage of preclinical diastolic dysfunction and because of the different phenotypes of the illness [30].

**Obesity Phenotype:** Obesity, defined as an elevated body mass index (BMI; kg/m^2^) >30, is a recognized risk factor for new onset of HFpEF [31,32] since it is a major determinant of arterial stiffness, hypertension [33], and LV hypertrophy with increased risk of diastolic LV dysfunction [34]. In addition, this condition is associated with a 4-fold greater prevalence of OSAS, which contributes to the pathogenesis of HFpEF [35]. About 60–75% of patients with HFpEF have a high BMI (>30 Kg/m^2^) [36]. It has been shown that, in such patients, the activation of the renin-angiotensin-aldosterone axis and the sympathetic nervous system, the inflammatory adipocytokines, and the induction of oxidative stress lead to cardiac interstitial fibrosis with an increase in cardiac rigidity and, consequently, an augmentation of the necessary energy required for LV diastolic relaxation [37,38]. 

T2DM is strictly linked to obesity and to the development of HFpEF. The 49% of 5988 patients constituting the study sample of the EMPEROR-Preserved trial was affected by diabetes [39]. This group of patients often develop diabetic kidney disease and chronic kidney disease (CKD). Indeed, approximately 50% of patients with HFpEF suffer from CKD [40]. Brouwers et al. reported that HF directly affects kidney function and vice versa, while CKD worsens cardiac function [41]. The interplay between the heart and the kidneys, the similarities in their microvascular networks, and the coexistence of CKD and HF make the role for microvascular dysfunction in the onset and worsening of both diseases more likely [42].

**Pulmonary Hypertension Phenotype:** The main leading cause of pulmonary venous hypertension (type 2) is increased pressure in the left atrial. In HFpEF, the right ventricular structure and function deteriorate over time; such deterioration is associated with coronary heart disease, obesity, atrial fibrillation, increased pressure pulmonary venous, and increased left heart filling pressures. When the left ventricular filling pressures increase, there is an increased afterload of the right ventricle, resulting in a remodelling of the pulmonary vascular system. Therefore, it becomes essential to carefully evaluate diuretic therapy in patients with HFpEF in an attempt to normalize pressures at the level of the left heart [43]. 

**Ischemic Heart Disease Phenotype:** In contrast to HFrEF, where obstructive coronary artery disease (CAD) is a significant illness determinant and needs specific management, ischemic myocardial injury does not play an essential role in the pathophysiology of HFpEF [44,45]. Though abnormal relaxation is the first mechanical manifestation of myocardial ischemia [46,47], acute coronary syndromes seldom are the cause of HFpEF [47,48]. Although the prevalence of CAD is high in HFpEF patients, non-invasive testing fails to detect the presence of CAD in at least one-third of HFpEF patients. Gerber et al. showed a temporal shift in the mix of types of HF in patients experiencing an acute CV event, with a recent increase in both midrange ejection fraction (HFmrEF) and HFpEF cases compared to HFrEF cases, which were more common in the past [49]. In a recent article, Elgendy and Pepine underlined that, aside from the traditional afterload–overload concept in the pathophysiology of HFpEF, coronary microvascular dysfunction, which is secondary to multiple co-morbidities, is an important pathogenetic mechanism [50]. It is, thus, essential to perform specific tests in the cath lab to identify this subset of patients to prevent and adequately treat ischemic and HFpEF symptoms. 

The integration of clinical data, biomarkers, and electrocardiographic and echocardiographic parameters is necessary to set up a correct therapeutic strategy for the whole spectrum of patients with HFpEF. Such integration is very complex; therefore, ways of phenotyping guided by mathematical/statistical algorithms, the so-called “machine learning”, of which a “cluster” analysis was first proposed by Shah et al. in 2015, represent the first example for outlining pheno-groups that may be a therapeutic target for randomized clinical trials in the future [51]. For example, the combination of SGLT2-Is and GLP-1 RAs, according to the available evidence, is indicated in diabetic patients with atherosclerotic disease and HF or at high risk of HF. In all other categories of patients, further studies are needed [19].

## 3. Cardiovascular Effects of GLP-1 RAs in T2DM 

The identification of glucagon-like peptide-1 (GLP-1), belonging to the family of gut-derived incretin hormones, paved the way years ago to the development of GLP-1 RAs, with the intention of enlarging the armamentarium of therapeutic options for T2DM. This new class of drugs, which is initially made up of short-acting compounds (exenatide, liraglutide, and lixisenatide) and subsequently includes new second-generation molecules that are administered once weekly (semaglutide, exenatide LAR, albiglutide, and dulaglutide), has shortly been demonstrated to have several vascular actions going beyond the mere anti-hyperglycemic effects. Consequently, a number of experimental and clinical studies have been conducted over time, aiming at better understanding the pathophysiological mechanisms underlying the beneficial CV effects (mainly on atherosclerotic CV disease) observed in some dedicated CV outcomes trials with GLP-1 RAs [52,53,54,55,56]. Indeed, with the exception of lixisenatide, all other GLP-1 RAs showed a trend toward a reduced incidence of MACEs (i.e., time to first event of either CV death or non-fatal myocardial infarction or stroke), which was significant in most of these trials, prompting a revision to the place of this class of drugs in the treatment algorithms of professional societies [57,58]. Of note, all of these CV outcome trials did aim at glycemic equipoise (that means having as much as possible similar glycemic control between the active drug and the placebo treatment); therefore, the glycated hemoglobin (HbA1c) reduction obtained with GLP-1 RA during the treatment period did not appear to be a plausible explanation for the observed CV benefits. This is also in accordance with the findings of previous large randomized clinical trials, which failed to demonstrate a clear effect of intensive glucose control as expressed by a marked HbA1c reduction on the CV outcomes and mortality in patients with T2DM [59]. As such, apart from glycemic control, other recognized clinical benefits of GLP-1 RAs have been highlighted, including reduction in weight and adiposity, improved blood pressure, and post-prandial excursions of triglycerides and apolipoprotein B, and perhaps reduction in some other diabetes-associated complications (for instance, renal disease) [60,61]. 

At the pathophysiological level, since GLP-1 RAs are expressed in endothelial cells, as well as in monocytes, macrophages, and vascular smooth muscle cells, native GLP-1 exerts a number of diverse actions on the vasculature [62]. As a matter of fact, enhancing the GLP-1 signaling by the administration of GLP-1 RAs can translate into significant beneficial effects on the diverse mechanisms contributing to the atherosclerotic process. GLP-1 RAs reduce oxidative stress by decreasing ROS production in cardiomyocytes and by decreasing the circulating levels of 8-iso prostaglandin F2α as well as carotid intima media thickness in T2DM patients [63]. In turn, the reduced exposure to ROS has been shown to slow down the transformation of monocytes/macrophages in foam cells [64,65], inhibit caspase-mediated apoptosis of foam cells, and reduce necrosis in the core of atherosclerotic plaques [66,67,68]. GLP-1 RAs also reduce monocyte accumulation in the vascular wall by affecting the expression of adhesion molecules, such as VCAM-1, MCP-1, E-selectin, and ICAM-1, which are driven by oxLDL-mediated activation of monocytes and macrophages in the early-stage atherosclerotic plaque [69,70]. Under GLP-1 receptor stimulation, monocytes preferentially give origin to M2 macrophages, instead of M1 macrophages, while the otherwise suppressed formation of Krüppel-like Factor 2 (KLF-2) becomes enhanced [71,72]. Endothelial cells cultured with GLP-1 RAs have been shown to express more eNOS, produce more NO, and suppress endothelin formation [73,74], resulting in overall increased vascular smooth muscle relaxation and endothelium-dependent vasodilation. Vascular smooth muscle proliferation and migration into the atherosclerotic plaque are additional pathophysiological processes potentially affected by GLP-1 RA administration [75,76].

Accordingly, animal models of accelerated atherosclerosis have confirmed the diverse beneficial effects of GLP-1 RAs on plaque progression, including reducing plaque hemorrhage, preserving the integrity of the fibrous cap, and preventing plaque rupture [77]. Noteworthy, these overall actions of GLP-1 RAs against systemic inflammation, vascular damage, and atherosclerosis have been replicated in several experimental studies with patients with metabolic syndrome and T2DM [78]. In addition to these findings, another intriguing hypothesis for the anti-atherosclerotic effects of GLP-1RAs is the reduction of epicardial adipose tissue [79], namely the metabolically active visceral fat depot of the heart which turns out to be an additional modifiable risk factor because of its anatomical and functional contiguity with the myocardium and coronary arteries.

Taken together, all this evidence supporting the significant role of GLP-1 receptor stimulation against the atherosclerotic process provides solid grounds for the preferential use of GLP-1 RAs in diabetic patients with or at high risk of atherosclerotic CV diseases. New research is on the way to explore the potential synergistic (additive?) effects of GLP-1 RAs with other classes of drugs with proven CV benefits, such as SGLT-2 Is, in an attempt to further improve CV protection in patients with T2DM (Table 1).

## 4. The SGLT2-Is Effects on Cardio-Renal Axis

Sodium-glucose cotransporter 2 belongs to a family of glucose transporter proteins localized in the first segment of the proximal renal tubule and responsible for the majority of filtered glucose and sodium reabsorption. SGLT2-Is are a novel class of drugs for the treatment of T2DM and HF [80,81,82].

Maximal glucose tubular transport (TmG) in healthy adult men is approximately 375 mg/min, which corresponds to 300 mg of glucose per day [83,84,85]. Because glucose filtration rate is lower than TmG under normoglycemic and hypoglycemic conditions, filtered glucose is completely reabsorbed in proximal convoluted tubules by SGLT2 receptor; conversely, glycosuria occurs when glycemia exceeds the threshold of 180 mg/day.

In patients with T2DM, the expression of SGLT2 receptors in the proximal tubule is higher than healthy individuals [86]. During hyperglycemia, as glucose and sodium reabsorptions are coupled, increased glucose reabsorption leads to increased sodium reabsorption, resulting in an increase in blood pressure; atrial natriuretic peptide release; glomerular afferent arteriole vasodilation mediated by nitric oxide, adenosine, and prostanoids; and efferent arteriole vasoconstriction through RAAS activation and angiotensin II action [87]. This results in an increased endoglomerular filtration pressure with glomerular hyperfiltration, which damages the mesangial structure and causes inflammation, fibrosis, renal damage, and albuminuria [88]. SGLT2-Is reduce sodium–glucose reabsorption in the proximal convoluted tubule, increasing diuresis, natriuresis, and glicosuria; the increased sodium concentration in the filtrate that reaches the macula densa implements afferent arteriole vasoconstriction and efferent arteriole vasodilation, lowering filtration pressure and restoring the normal production of filtrate [89]. Moreover, SGLT2-Is reduce active tubular transport work by decreasing renal oxygen demand [90]; improve mitochondrial function and autophagy [91]; and reduce oxidative stress, apoptosis, tubule-interstitial inflammation, and fibrosis [92]. Empaglifozin protects against renal ischemia-reperfusion injury in mice by attenuating tubular damage, reducing proinflammatory cytokine expression, and inhibiting apoptosis [93]. In mice with diabetic kidney disease, Empaglifozin improves kidney morphology and function by reprogramming the protein and metabolic profile, including renal reductive stress regulation and renal mitochondrial dysfunction and oxidative stress reaction reduction [94]. Finally, it increases hypoxia-inducible factor 2 alfa, stimulating erythropoiesis and improving renal oxygenation [95]. Hematocrit increase may favorably influence cardiomyocyte mitochondrial function, angiogenesis, cell proliferation, and inflammation; in addition, it directly enhances myocardial tissue oxygen delivery. Mazer et al. [96] recently evaluated this in the EMPA-Heart CardioLink-6 randomized clinical trial and demonstrated that EPO levels increased significantly after one month of Empagliflozin treatment in people with T2DM and coronary artery disease, and was accompanied by an increase in hematocrit and reduced ferritin and red blood cell haemoglobin.

CV beneficial effects of SGLT2-Is include positive hemodynamic effects with a reduction in preload and afterload and improved cardiac contractility, related not only to the increased diuresis and natriuresis, but also to nephron remodeling, endothelial function improvement and, through glycosuria, also loss of body weight and adipose tissue with reduced insulin resistance [87,97,98]. A systematic review of preclinical studies showed that SGLT2-Is attenuated vascular dysfunction through a combination of mechanisms that appear to act independently of glucose-lowering benefits [99]. SGLT2-Is reduce blood pressure also in the absence of an increased heart rate, suggesting that these agents may lead to a reduction in sympathetic nervous system (SNS) activity [100,101]. As HF progresses, a continual decline in mitochondrial oxidative metabolism occurs; SGLT2-Is were shown to increase circulating ketone levels, secondary to mobilizing adipose tissue fatty acids, which are then used by the liver for ketogenesis [102]. These ketones have been proposed to improve cardiac energetics and cardiac efficiency [103,104].

Current clinical practice guidelines strongly recommend the use of SGLT2-Is in the treatment of patients with HFrEF (Class I). However, these guidelines still make a weaker recommendation for their use in HF with mildly reduced or preserved ejection fraction, based on the data of the DELIVER that have been published recently [105]. A recent comprehensive meta-analysis by Vaduganathan et al. built the evidence base for the benefit of SGLT2-Is on the hospitalization for HF death, and in patients with preserved or mildly reduced ejection fraction [106]. These authors did a prespecified meta-analysis of the DELIVER and the EMPEROR-Preserved, and subsequently included trials that enrolled patients with reduced ejection fraction (DAPA-HF and EMPEROR-Reduced) and those admitted to the hospital with worsening HF with any ejection fraction (SOLOIST-WHF), for a combined meta-analysis providing power to assess various clinical outcomes. SGLT2-Is reduced composite CV death or first hospitalization for HF, CV death, first hospitalization for HF, and all-cause mortality; these results were consistently observed across all five trials, supporting the role of SGLT2-Is as a fundamental therapy for HF irrespective of ejection fraction or care setting.

**Figure 2:**
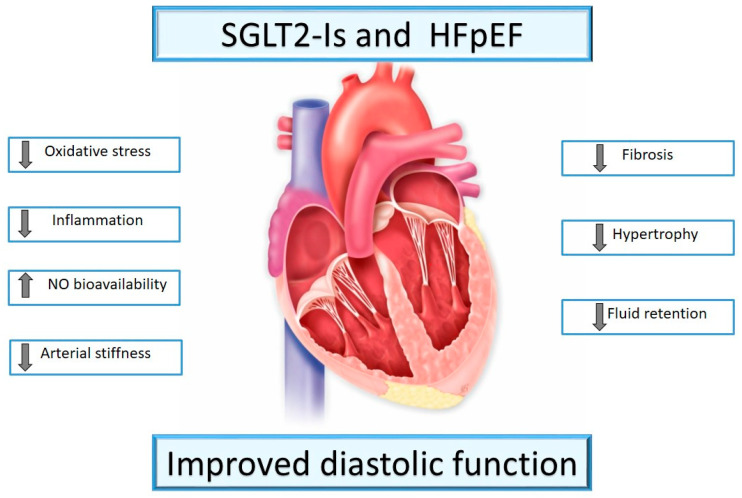
SGLT2-Is and HFpEF. SGLT2-Is improve left ventricular diastolic function.

In clinical trials, SGLT2-Is reduced the risk of dialysis, transplantation, or death due to kidney disease in individuals with T2DM and also provided protection against acute kidney injury with consistent benefits across studies. These data provide substantive evidence supporting the use of SGLT2-Is to prevent major kidney outcomes in people with T2DM. The trials that determined these conclusions included the EMPA-REG OUTCOME for Empagliflozin, the CANVAS Program and CREDENCE for Canagliflozin, and the DECLARE–TIMI 58 for Dapagliflozin. Although there was evidence that the proportional effect of SGLT2-Is might attenuate with declining kidney function, there was clear, separate evidence of benefit for all estimated glomerular filtration rate (eGFR) subgroups, including in participants with a baseline eGFR 30–45 mL/min per 1.73 m^2^. Reno-protection was also consistent across studies, irrespective of baseline albuminuria and use of RAS blockade [107]. As it has been proven by large, double-blind clinical trials, Empagliflozin not only decreases HbA1c in diabetic patients, but also improves their life expectancy by reducing CV mortality [108]. Canagliflozin, Dapagliflozin, and Sotagliflozin significantly decrease the composed primary end-point, including CV mortality and other CV outcomes [109]. Ertugliflozin shows non-inferiority vs. placebo in reducing CV mortality and other CV Outcomes [110]. The exact mechanism has not been fully established yet, and SGLT2-Is show many additional beneficial effects which contribute to their wider use, even in non-diabetic patients [111] (Figure 2).

## 5. The Rationale for the Use of GLP-1RAs and SGLT2-Is in T2DM Patients with HFpEF 

Empagliflozin in DM patients with CV disease leads to a reduction in LV mass and LV end-diastolic volume, as well as to an improvement in LV diastolic function after a period of three to six months of therapy [112]. 

There are several possible mechanisms that may explain SGLT2-Is-induced LV mass reduction. Vasilakou et al. showed that Dapagliflozin could mediate the regression of LV mass by reducing SBP. In fact, there was a statistically significant correlation between the ambulatory reduction of SBP and LV mass reduction that could support this plausible mechanism. It has been observed that SGLT2-Is lead to a reduction of SBP in the range of 3–5 mmHg in patients with T2DM [113]. Another mechanism that can explain this phenomenon is given by the reduction of the ventricular preload due to osmotic diuresis and natriuresis. The increase in diuresis could lead to an improved ventricular load condition by reducing the LV wall stress [114].

The analysis of the EMPA-REG OUTCOME study suggested that approximately 50% of the CV benefit could be attributed to Empagliflozin-induced hemoconcentration [115]. Weight loss is most significant in the first 4–6 months of therapy and is likely due to glucose excretion and associated calorie loss [116]. Furthermore, SGLT2-Is-induced glycosuria improves β cell function and insulin sensitivity, resulting in LV mass regression [117].

Verma et al. reported the effects of a 3-month Empagliflozin (10 mg/day) treatment in terms of heart function. In 10 patients with T2DM and established CV disease, treatment with Empagliflozin was associated with a significant reduction in LV mass index and improved LV diastolic function, as assessed in terms of lateral e′ (8.5 vs. 9.6 cm/s, *p* = 0.002) [118]. However, the exact mechanism of the improvement in diastolic function may require further studies. 

A small study that evaluated the effect of Canagliflozin on LV remodeling in DM patients with HF showed that LV filling pressure, as assessed using a tissue Doppler, decreased significantly after 6 months of treatment, and the effect was maintained even after 12 months of therapy. Many other mechanisms may be responsible for the improvement of LV diastolic function. From those, the reduction of preload, the improvement of endothelial dysfunction and aortic stability, the reduction of epicardial fat accumulation, and the hypertrophy of visceral adipocytes are very important [119,120,121].

**Figure 3:**
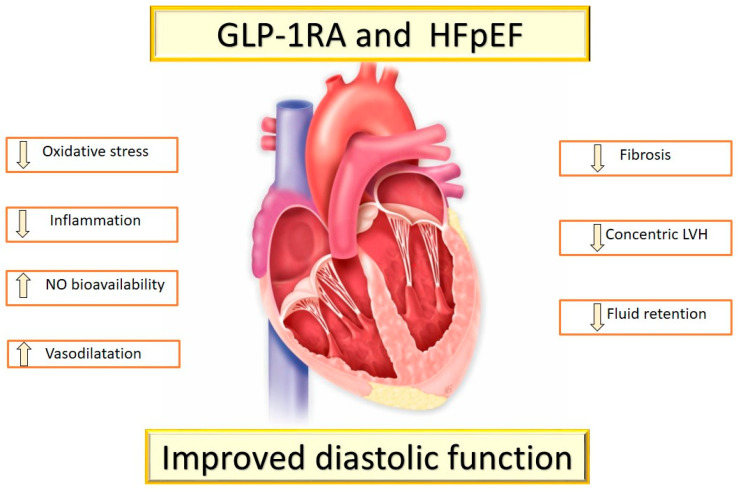
GLP-1 RA and HFpEF. GLP-1 RA improves left ventricular diastolic function.

GLP-1 RAs also have positive effects on LV diastolic function. Bizino et al. demonstrated that the administration of liraglutide reduced early LV diastolic filling and LV filling pressure [122]. GLP-1 RA-induced weight loss might improve LV diastolic function [123] (Figure 3). In addition, a direct cardio-protective effect of GLP-1 RA therapy has been demonstrated in preclinical studies, and in some but not all human studies [124].

Coenraad Withaar et al., in a multi-hit mouse model of HFpEF showed that treatment with liraglutide reduces cardiometabolic dysregulation; improves cardiac function; reduces myocardial hypertrophy and fibrosis; and reduces atrial volume, natriuretic peptide levels, and pulmonary congestion. Although it is not yet clear what is the true cardioprotective mechanism of GLP-1 RAs in diabetic patients with HFpEF or in diabetic patients at high risk of HFpEF, the authors are in agreement with the hypotheses that have been previously formulated in other experimental and clinical studies: the beneficial effects on the heart arise not only from a reduction in body weight, LV mass, and cardiac stiffness; but they also come from the direct effects of GLP-1 RAs on the CV system deriving from natriuresis, the central and peripheral vasodilation, and a reduction in both blood pressure and cardiac fibrosis. GLP-1 RA may postpone the onset of HFpEF as it may delay the onset of diabetic cardiomyopathy [7].

## 6. Discussion 

HFpEF is a multifactorial disease with different phenotypic aspects; therefore, due to the high complexity of this syndrome, therapies targeting the sympathetic and RAA system activation have not shown mortality benefit in HFpEF. The pharmacological treatment of HFpEF represents the biggest ‘unmet medical need’ in the scientific community, and not only in the CV community. In recent years, various consensus documents have outlined the different modalities applicable to the phenotyping of patients with HFpHF. This underscores how the “one size fits all” is not suitable for patients with HFpEF, and it is necessary for therapeutic strategies to be individualized through the identification of the clinical phenotypes of the disease. Currently, therapies for HFpEF are often limited to something to improve the symptoms.

People affected by diabetes have a high risk of developing HFpEF and vice versa, suggesting shared pathophysiological mechanisms exist; this, in turn, engenders the potential for common treatments. In fact, T2DM is closely associated with microvascular complications and microvascular dysfunctions, and plays an important role in the onset of HFpEF.

SGLT2-Is, which started as antidiabetic drugs, have become one of the key pillars of HFrEF therapy due to their positive pleiotropic effects on the kidney, liver, pancreas, blood vessels, and adipose tissue. 

The EMPA-REG OUTCOME showed that Empagliflozin treatment of DM patients had no significant effect on hemodynamic parameters during the three months of therapy, but induced rapid and sustained improvement in LV diastolic function (E/e′) [112]. Proscribed SGLT2-Is therapy after acute myocardial infarction was not associated with improved longitudinal LVEF or LV strain, but was correlated with favourable changes in diastolic function parameters. The IDDIA showed that Dapagliflozin, in addition to standard anti-hyperglycemic therapy, in patients with T2DM was related to a significant improvement in LV diastolic dysfunction, as assessed by diastolic stress echocardiography, compared to placebo [125].

The use of Dapagliflozin resulted in a significant reduction in LV filling pressure as estimated by E/e′ during exercise in patients with type 2 DM.

The EMPEROR-Preserved was the first study on HFpEF that compared Empaglifozin vs. placebo in patients with EF > 40%, regardless of the presence of diabetes, and demonstrated a reduction in the risk of HF-related hospitalizations and CV mortality and an improvement in renal outcomes. In fact, previous studies, such as the CHARM-Preserved (with candesartan), the TOPCAT (with spironolactone), and the PARAGON HF (with sacubitril/valsartan), did not document a significant treatment benefit over placebo in this clinical setting. The EMPEROR-Preserved trial is a real revolution in the treatment of HFpEF because, after years of inconclusive studies of drugs used in such condition, its results offer for the first time the potential benefit of improving outcomes and quality of life in HFpEF patients [126].

The recent DELIVER trial demonstrated that Dapagliflozin is effective compared to placebo in reducing CV death and HF hospitalizations in patients with mildly reduced or preserved ejection fraction, and in patients with previous <40% EF which subsequently increased above 40% [105], opening a new horizon in the treatment of HFpEF. 

Other antidiabetic drugs that have demonstrated positive CV effects are GLP-1 RAs. In previous randomized controlled trials with patients with T2DM, liraglutide, semaglutide, and duraglutide have been shown to reduce CV mortality, but not the incidence of HF or hospitalization for HF. A growing body of evidence shows a significant beneficial effect of GLP-1 RAs on LV diastolic function in patients with T2DM. Studies that examined patients with T2DM and without CV disease demonstrated that liraglutide reduces LV filling pressure [127] and improves LV diastolic function, while LV diastolic function is not improved by sitagliptin and linagliptin. A meta-analysis study that included 592 patients treated with different oral antidiabetic drugs (sitagliptin, linagliptin, pioglitazone, rosiglitazone, voglibose, and glimepiride), showed that only liraglutide was associated with significant improvement in LV diastolic function [128]. These results are encouraging and warrant further investigation in subjects with HFpEF, since, to date, there are no randomized studies with GLP-1 RAs in this setting of HF.

## 7. Conclusions

SGLT2-Is and GLP-1 RAs could be important weapons in the therapy of HFpEF, but to date, the data are poor because there is still a lack of studies evaluating the effects of their combined treatment. The goal should be to provide unambiguous clinical evidence in order to improve the effectiveness of the therapeutic armamentarium for the prevention and treatment of CV and renal diseases in cardio-metabolic high-risk patient, in order to improve organ damage and reduce CV morbidity and mortality. Therefore, what is the optimal therapy for HFpEF? Certainly, it is crucial to treat the cause of HFpEF and control all comorbidities, not only with medical therapy but also with changes in lifestyle habits. A future challenge will be to try to tailor an appropriate treatment strategy specific to the phenotype-dominant disease in individual patients.

## Figures and Tables

**Table 1:** Potential biological effects of SGLT1-Is or GLP-1 RAs, alone or in combination therapy.

	Agent	SGLT2 Inhibitors	GLP-1 Agonists	Combination Therapy
Effects				
Oxidative stress		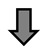	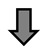	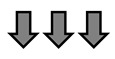
Inflammation		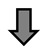	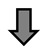	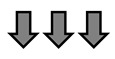
Endothelial dysfunction		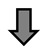	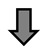	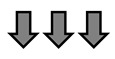
Natriuresis		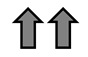	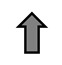	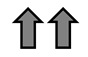
Diuresis		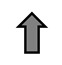	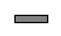	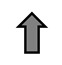
Plaque stability		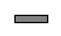	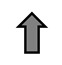	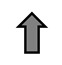
Blood pressure		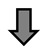	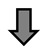	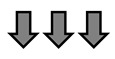
Body weight		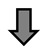	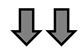	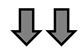
Lipid profile		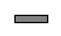	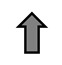	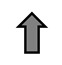
Epicardial fat		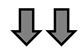	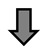	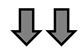
Myocardial fibrosis		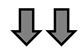	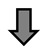	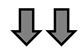
Insulin resistance		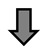	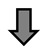	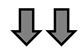
Beta cell function		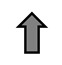	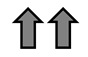	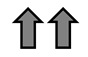

## Data Availability

No new data were created or analyzed in this study. Data sharing is not applicable to this article.
